# Utilizing MRI, [^18^F]FDG-PET and [^89^Zr]Zr-DFO-28H1 FAP-PET tracer to assess inflammation and fibrogenesis in a reproducible lung injury rat model: a multimodal imaging study

**DOI:** 10.3389/fnume.2023.1306251

**Published:** 2023-12-12

**Authors:** Milou Boswinkel, René Raavé, Andor Veltien, Tom WJ Scheenen, Nina Fransén Petterson, René in ‘t Zandt, Lars E. Olsson, Karin von Wachenfeldt, Sandra Heskamp, Irma Mahmutovic Persson

**Affiliations:** ^1^ Department of Medical Imaging, Radboud University Medical Centre, Nijmegen, Netherlands; ^2^ Truly Labs, Medicon Village, Lund, Sweden; ^3^ Lund University BioImaging Centre, Faculty of Medicine, Lund University, Lund, Sweden; ^4^ Department of Translational Medicine, Medical Radiation Physics, Lund University, Malmö, Sweden; ^5^ Department of Hematology, Oncology and Radiation Physics, Skåne University Hospital, Malmö, Sweden

**Keywords:** bleomycin, imaging, fibroblast activating protein (FAP), magnetic resonance imaging (MRI), positron emission tomography (PET), reproducibility experiments, animal models, lung fibrosis

## Abstract

**Objective:**

Accurate imaging biomarkers that indicate disease progression at an early stage are highly important to enable timely mitigation of symptoms in progressive lung disease. In this context, reproducible experimental models and readouts are key. Here, we aim to show reproducibility of a lung injury rat model by inducing disease and assessing disease progression by multi-modal non-invasive imaging techniques at two different research sites. Furthermore, we evaluated the potential of fibroblast activating protein (FAP) as an imaging biomarker in the early stage of lung fibrosis.

**Methods:**

An initial lung injury rat model was set up at one research site (Lund University, Lund, Sweden) and repeated at a second site (Radboudumc, Nijmegen, The Netherlands). To induce lung injury, Sprague-Dawley rats received intratracheal instillation of bleomycin as one single dose (1,000 iU in 200 µL) or saline as control. Thereafter, longitudinal images were acquired to track inflammation in the lungs, at 1 and 2 weeks after the bleomycin challenge by magnetic resonance imaging (MRI) and [^18^F]FDG-PET. After the final [^18^F]FDG-PET scan, rats received an intravenous tracer [^89^Zr]Zr-DFO-28H1 (anti-FAP antibody) and were imaged at day 15 to track fibrogenesis. Upon termination, bronchoalveolar lavage (BAL) was performed to assess cell and protein concentration. Subsequently, the biodistribution of [^89^Zr]Zr-DFO-28H1 was measured *ex vivo* and the spatial distribution in lung tissue was studied by autoradiography. Lung sections were stained and fibrosis assessed using the modified Ashcroft score.

**Results:**

Bleomycin-challenged rats showed body weight loss and increased numbers of immune cells and protein concentrations after BAL compared with control animals. The initiation and progression of the disease were reproduced at both research sites. Lung lesions in bleomycin-exposed rats were visualized by MRI and confirmed by histology. [^18^F]FDG uptake was higher in the lungs of bleomycin-challenged rats compared with the controls, similar to that observed in the Lund study. [^89^Zr]Zr-DFO-28H1 tracer uptake in the lung was increased in bleomycin-challenged rats compared with control rats (*p* = 0.03).

**Conclusion:**

Here, we demonstrate a reproducible lung injury model and monitored disease progression using conventional imaging biomarkers MRI and [^18^F]FDG-PET. Furthermore, we showed the first proof-of-concept of FAP imaging. This reproducible and robust animal model and imaging experimental set-up allows for future research on new therapeutics or biomarkers in lung disease.

## Introduction

Imaging is increasingly employed in the diagnostics and follow-up of treatment in various types of fibrotic lung diseases (1–3). The detection of fibrosis progression at an early stage is essential for the optimal mitigation of symptoms and disease. With continuous technical development of different imaging modalities, as well as increasingly automated workflows of image processing, new imaging biomarkers for lung disease are expected to emerge (4, 5). Therefore, it is of great importance to have reproducible animal models that can be replicated by scientists globally, across different labs.

Animal models of lung injury, mimicking human pathology, can be induced by various compounds and different exposure regimes. The most commonly used agent for the induction of lung injury is bleomycin, by administration directly into the lung as an intratracheal (i.t.) instillation (6–8). Bleomycin is a chemotherapeutic drug, mainly employed in the clinical setting for the treatment of testicular cancer and Hodgkin's lymphoma, and is well-known for its toxic effects, including lung injury (9–11). When bleomycin is used to induce lung injury in animal models, inflammation will be initiated followed by fibrotic scar formation (12, 13). These dynamics in disease transition and progression can be used to study various types of pathologies and imaging biomarkers.

Having a robust lung injury model that is easily reproducible is key in the development of disease biomarkers. In particular, non-invasive and longitudinal follow-up imaging studies are preferred to track disease progression (4, 14, 15). Employing magnetic resonance imaging (MRI) to assess lung disease has increasingly been explored. MRI offers the flexibility of testing different sequences and exploring contrast mechanisms in order to detect lesions of different properties. Depending on the existing pathology, MRI can be used to assess inflammation and edema as well as fibrosis in the lung tissue, hence favoring longitudinal studies with a robust workflow that can be achieved in the search for unique imaging biomarkers during disease progression (16–18).

Typically, the initial bleomycin-induced inflammation in the lung results in increased energy demand within the inflamed lung areas (19). This alteration of metabolism can be tracked by the most commonly used radiotracer in positron emission tomography (PET) imaging: the synthetic glucose molecule fludeoxyglucose coupled to radioactive fluorine ([^18^F]FDG) (20–22). Once the initial inflammation ceases, wound-healing processes, such as fibrogenesis, are initiated. During fibrogenesis, fibroblasts will be induced (23–25), along with the increased production of components of the extracellular matrix (ECM), e.g., collagens, glycoproteins, fibronectin, and proteoglycans (13, 26, 27). Previously explored imaging biomarkers in the bleomycin model involved the assessment of lesions using MRI during inflammation and fibrosis, as well as PET tracers mapping disease progression in terms of increased uptake of [^18^F]FDG and Collagen type I tracer uptake within the lung (28–35). For tracking early-onset fibrosis, new emerging radiotracers for fibroblast activating protein (FAP) could be used to track the presence of activated fibroblasts in tissue (25, 36). The antibody 28H1 specifically binds FAP, and when radiolabeled, it can be used to visualize fibrotic processes (37, 38). Therefore, it may be a reliable tool to study the onset of fibrogenesis at an early stage of lung disease.

Here, we aim to (1) reproduce the animal model of intratracheal exposure with bleomycin for the induction of lung injury in rats at another research site, (2) reproduce imaging biomarkers using MRI and [^18^F]FDG for the assessment of disease progression, and (3) match the imaging data processing from scans generated at two different research sites. Furthermore, using the reproduced set-up, we explored the potential of a novel imaging biomarker to track early fibrogenesis by targeting fibroblast activation using radiolabeled 28H1.

## Materials and methods

### Ethical approval, animals, and experimental set-up

The initial animal experiments were performed at Lund University, Lund, Sweden, and were reviewed and approved by the Ethical Committee of Lund/Malmö, Sweden (permit numbers 4003/2017 and 3226/2017). Experimental details for these studies are published and described in detail elsewhere (29). In the present study, parts of the data from the published study (29), i.e., experiments previously performed by the research site in Lund, are presented within the results section where they are compared to the new data generated at Radboudumc, Nijmegen, the Netherlands.

The animal experiments were then reproduced at the second research site at Radboudumc and were approved by the Dutch central committee on animal research and the local ethical committee on animal research of the Radboudumc (protocol number 2017-0028). All animal experiments were performed according to the local institutional guidelines and reported following the ARRIVE guidelines (39). Therefore, the experimental details below only describe the procedures performed at Radboudumc, unless stated otherwise. Studies at Radboudumc were matched as closely as possible to the Lund study with regard to animal welfare, housing conditions, imaging protocols, termination procedures, and histology assessment. Table 1 shows the main similarities and discrepancies between the experimental procedures between the two sites.

**Table 1:** Summary of techniques and methods used to reproduce the lung injury model in rats, imaging biomarkers and data processing, at both research sites.

Comparing parameter		Sites	
		Nijmegen	Lund
Bleomycin	Manufacturer	SIGMA-ALDRICH® Lot: B5507-15UN	SIGMA-ALDRICH® Lot: B5507-15UN
	Administration route	i.t.	i.t.
	Dose	1,000 iU	1,000 iU
MRI	Vendor	Bruker	Bruker
	Field strength	7T	9.4T
	Sequence	FLASH	UTE
	Echo (TE)	0.71 ms	1.0 ms
[^18^F]FDG-PET	Vendor	Siemens	Nano-PET Mediso
	Tracer activity	30 MBq	30 MBq
	Acquisition time	20 min	20 min
	Reconstruction parameters	OSEM	MLEM
	Attenuation correction	Transmission scan	CT scan
PET image processing	Software	VivoQuant^TM^ (InVicro)	VivoQuant^TM^ (InVicro)
Histology	Staining technique	H&E	H&E
		Masson's-trichrome^a^	Masson's-trichrome
		FAP (immunohistochemistry)	—
	Quantification method	Modified Ashcroftscore^a^	Modified Ashcroft score
BAL analysis	Cells	Total counts	Total counts
		Differential counts^a^	Differential counts
	Supernatant	Total protein concentration	Total protein concentration
Additional methods	RT-qPCR	—	Gene profiling from lung tissue homogenates
	*In vivo* imaging	[^89^Zr]Zr-28H1 PET of the lung	—
	*Ex vivo* tracer uptake	Autoradiography (lung) and *ex vivo* biodistribution	—

^a^ These methods were performed at the Nijmegen research site, but quantification was performed by the team in Lund.

At Radboudumc, male Sprague-Dawley rats (Envigo, Horst, the Netherlands, aged 6–8 weeks, weighing 250–350 g) were assessed daily for their general wellbeing. The animals had unlimited access to food and water in a controlled environment in individually ventilated cages, with the temperature regulated at 22°C ± 1 °C and a humidity of 55% ± 10%, with 12 h dark/light cycles. Rats were acclimatized for ≥7 days before any experimental procedure and were randomly allocated to the two experimental groups receiving either saline or bleomycin instillation via the i.t. route. Soft wet food was provided inside the cage for the animals exposed to bleomycin to prevent severe body weight loss. This was replaced by normal chow when the weight of the animals stabilized.

**Figure 1:**
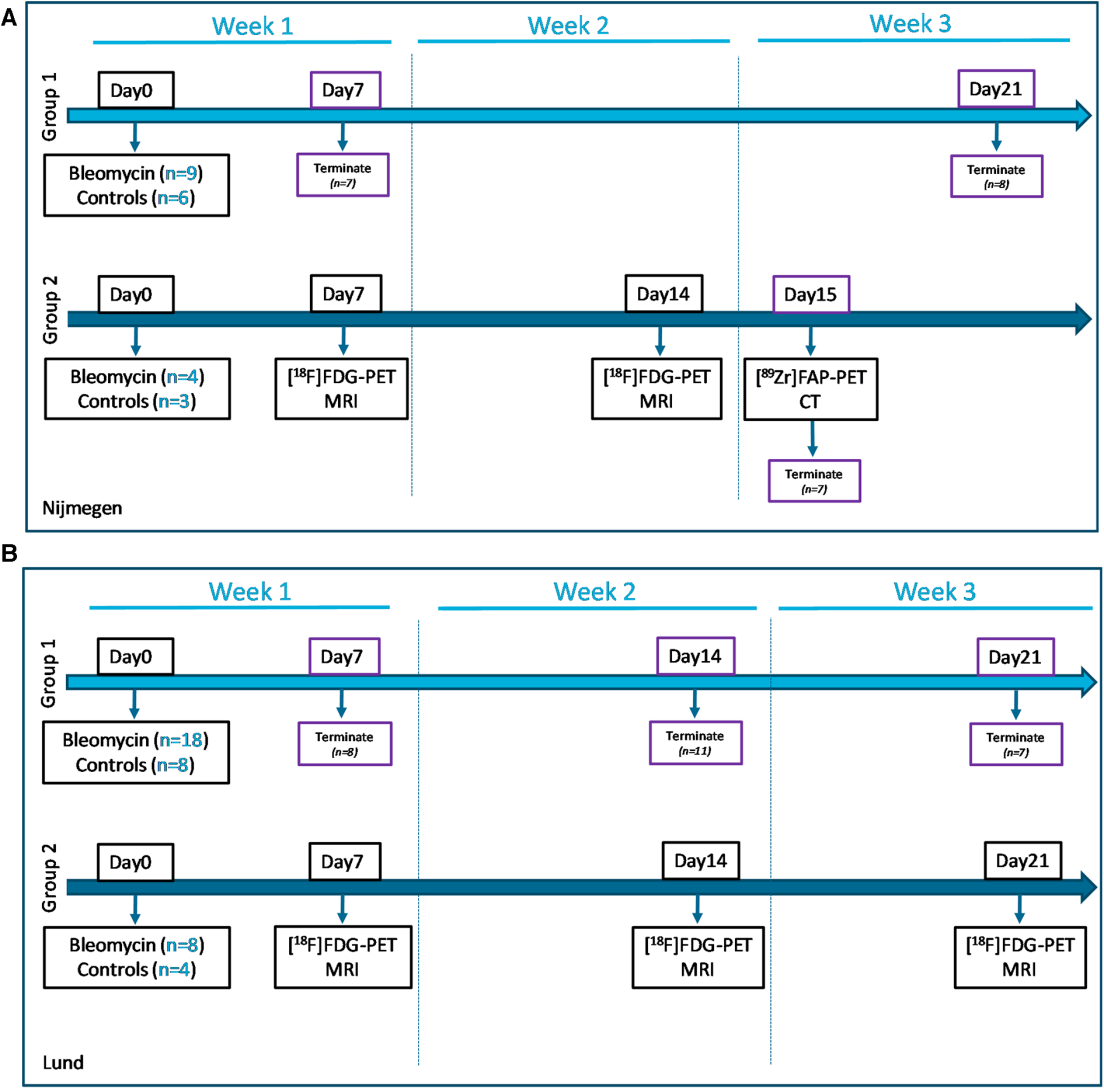
Study layouts of the workflow during imaging and sample collection. (**A**) Study conducted at the Nijmegen site, with animals from Group 1 being terminated at day 7 (week 1) and day 21 (week 3), for model characterization and confirmation of reproducibility, while Group 2 was imaged longitudinally for 2 weeks. (**B**) The study layout of the Lund study included Group 1 with terminal sampling at time points week 1, 2 and 3 postinstillation. The purple boxes indicate termination days. Similarly, longitudinal imaging was performed in animal Group 2 at the research site Lund, using multimodality approach with combined MRI and PET/CT.

The experimental set-up is illustrated in Figure 1A. In Group 1, the model was characterized by terminal sample collections at days 7 and 21 after the induction of lung injury. Group 2 included the longitudinal imaging, where animals were imaged at days 7, 14, and 15 after instillation. Imaging sessions were performed using MRI, PET, and computed tomography (CT). The animal groups from the experiments at the Nijmegen site corresponded to the groups in the Lund study (Figure 1B).

### Bleomycin challenge

Bleomycin (Sigma Aldrich, St. Louis, MO, USA) was administered as a single i.t. dose on day 0, with a concentration of 1,000 iU, dissolved in 200 µL saline. The control animals received the same volume of saline. The administration of bleomycin was performed on lightly sedated animals, which were placed in a supine position on a board with a decline of approximately 45°. The i.t. administration was performed using a syringe connected to a blunt cannula with a small steel marble at the top. Once the cannula passed the larynx, 200 µL of bleomycin was administered (or saline in control animals), followed by a 100 µL air puff immediately afterwards to increase distribution.

### FAP tracer conjugation and radiolabeling

Human IgG1 antihuman/mouse FAP antibody 28H1 (kindly provided by Roche; Basel Switzerland) was conjugated with p-SCN-Bn-deferoxamine (ITC-DFO; Macrocyclics, Plano, TX, USA) in 1 M NaHCO_3_, pH 9.5 using a 30-fold molar excess of ITC-DFO for 1 h on a shaker at room temperature. Unbound chelator was removed via dialysis (Slide-A-Lyzer cassette, MWCO 20000; ThermoScientific, Waltham, MA, USA) against 5 L of metal free phosphate-buffered saline (PBS; pH 7.4) containing 2 g/L Chelex (Bio-Rad, Hercules, CA, USA). DFO-modified 28H1 was radiolabeled with [^89^Zr]Zr(oxalate)_2_. First, the pH of [^89^Zr]Zr(oxalate)_2_ was adjusted to pH 7.4 using 2 M Na_2_CO_3_ before adding 0.5 M HEPES (pH 7.4). Subsequently, 28H1-DFO was added and incubated at 37 °C at 300 rpm for 1 h. The radiolabeling efficiency was determined using iTLC on silica gels chromatography strips (ITLC-SG; Agilent Technologies, Santa Clara, CA, USA) with 0.1 M sodium citrate buffer (Sigma Aldrich, St. Louis, MO, USA) as mobile phase. Radiolabeling efficiency exceeded 95% with a specific activity of 0.25 MBq/µg.

### Longitudinal imaging workflow

#### Experimental design and timeline

Rats assigned to Group 2 were imaged by [^18^F]FDG-PET and MRI at days 7 and 14. The workflow consisted of anesthetizing the animal, measuring blood glucose (Accu-Chek Aviva; Roche, Basel, Switzerland), and injecting 30 MBq of [^18^F]FDG intravenously in the tail vein. Subsequently, the anesthetized rat was transferred to a heated animal bed, followed by MRI and thereafter PET imaging at 60 min after the [^18^F]FDG injection. At day 14, after the [^18^F]FDG-PET and MRI scan session, rats were injected with tracer [^89^Zr]Zr-DFO-28H1 (5 MBq, 20 µg) and imaged by PET and CT the following day (Figure 1A). The animals were kept anesthetized during the scan sessions by providing 2%–3% isoflurane, using carrier gas O_2_ mixed with medical air (ratio 1:2). The different imaging modalities were combined in one workflow, constantly keeping the animal under anesthesia and in the same bed to facilitate coregistration. Rats were terminated after the last imaging session via an intraperitoneal overdose of pentobarbital sodium (Euthasol 20%; AST farma, Oudewater, NL).

#### Magnetic resonance imaging

MRI at Radboudumc was performed using a preclinical 7T MR system (ClinScan; Bruker BioSpin, Ettlingen, Germany) interfaced with a clinical user interface (Syngo VB15; Siemens Healthcare, Erlangen, Germany). Rats were positioned supine on a 4 × 1 array receive-only coil, which was combined with an 86 mm diameter transmit whole-body coil. Breathing was measured using an air cushion and breathing speed was maintained at approximately 60 breaths per minute. Body temperature was measured using a rectal fiber optical thermometer and maintained at 37 °C using heated air. 2D gradient echo images were acquired using the fast low angle shot (FLASH) sequence with a slice thickness of 1 mm, field of view of 64 mm × 64 mm and a matrix of 256 × 256, resulting in an in-plane resolution of 0.25 mm × 0.25 mm. Acquisition parameters were as follows: repetition time (TR) = 203 ms; 40 slices; flip angle (FA) = 25°; 16 averages; and echo time (TE) = 0.71 ms.

#### Positron emission tomography

PET imaging at Radboudumc was performed using an Inveon animal PET scanner (Siemens Preclinical Solutions, Erlangen, Germany). The [^18^F]FDG-PET acquisition time was 20 min and the [^89^Zr]Zr-DFO-28H1 PET acquisition time was 30 min. Transmission scans (5 min, Co-57 source) were acquired for all PET scans. Reconstruction was performed using the Inveon Acquisition Workplace software with an iterative 3D ordered subset expectation maximization using maximum *a priori* with shifted Poisson distribution algorithm with the following parameters: matrix = 256 × 256 × 161; pixel size = 0.4 mm × 0.4 mm × 0.8 mm; and a corresponding beta of 0.05 mm.

#### Computed tomography

After FAP imaging on day 15, CT imaging was performed using a U-SPECTII/CT (MILabs, Utrecht, The Netherlands) to facilitate MRI and PET image overlay. Animals were scanned in one bed position with a spatial resolution of 160 μm, 65 kV, and 0.615 mA. Scans were reconstructed with MILabs reconstruction software.

### Imaging data analysis

All acquired images were transferred to Lund University, where the DICOM raw-data files were uploaded onto the image analysis software platform VivoQuant^TM^ (InVicro v.2021). Images from different imaging modalities acquired at the same time point were coregistered. Subsequently, the 2D transverse slices from the MRI scans served as the template for segmentation of the lung region of interest (ROI) and was guided by the software tool Spline Tool for optimal segmentation flow. Further details on how the lung ROI was segmented are presented as an ROI atlas for each 2D slice (Supplementary Figure S1A). The MRI signal was processed according to the line intersect method where histogram analysis generated a signal threshold to identify the so-called high-signal vs. normal lung signal intensity (29), also illustrated by a schematic image in Supplementary Figure S1B. Image data from the same ROI were then processed further to assess total lung volume, lung MRI signal, and PET tracer uptake in the lung.

The [^18^F]FDG and [^89^Zr]Zr-DFO-28H1 uptake was presented as fraction uptake (% activity in the lung of the total injected activity).

### Termination and sample collection

After an overdose of pentobarbital sodium, heartbeat and breathing arrest occurred. Immediately thereafter, BAL was performed using PBS, which flowed into the lungs via tracheostomy tubing, for 2 min, followed by collection of the lavage fluid. This procedure was performed twice; thereafter, BAL fluid (BALF) was immediately put on ice. Subsequently, the right lung lobes were ligated off and the left lung was insufflated with 4% paraformaldehyde. The right lung lobes were dissected and snap frozen in −70 °C until use and the left lung lobe was fixated using 4% formalin for 3 days, before being embedded in paraffin blocks.

In Group 2, other relevant organs and tissues were collected to determine the biodistribution of [^89^Zr]Zr-DFO-28H1 *ex vivo*. Radioactivity was measured in the blood, muscle, heart, liver, spleen, pancreas, stomach, duodenum, kidney, knee, femur, and bone marrow.

### Analyzing BAL cells and supernatants

BALF was spun down (1,000 g, 10 min, at 4 °C) and supernatant was stored at −20 °C until analysis. The cell pellet was resuspended in 1 mL PBS and counted. The cell suspensions were diluted and approximately 50,000 cells were used to generate cytospins.

After drying, the cytospins were stained with May-Grünwald and Giemsa stain (Sigma Aldrich, St. Louis, MO, USA) and a differential count was performed. A minimum of 400 cells were counted from each sample replicate, and a mean value was generated from two to three replicates from each sample. The differential cell counts were performed by two persons independently, generating mean counts for each BAL sample.

The total protein concentration was determined in BALF using the Bradford Protein Assay kit from Bio-Rad (#5000006; Hercules, CA, USA), according to the manufacturer's instructions.

### *Ex vivo* biodistribution of [^89^Zr]Zr-DFO-28H1

Lung tissues were weighed and measured on a multichannel analyzer (MCA) with a well-type sodium iodide (NaI) crystal before BAL was performed. The other relevant organs (blood, muscle, heart, liver, spleen, pancreas, stomach, duodenum, kidney, knee, femur, and bone marrow) were dissected, weighed, and assessed for activity, using a *γ*-counter (Wizard; Perkin-Elmer, Waltham, MA, USA). The results are presented as a percentage of the injected activity per gram tissue (%IA/g).

### Histology staining and analysis

The left lung was sectioned (4 µm) in the sagittal plane at four different positions to obtain clear visualization of the widespread lesions throughout the whole left lung lobe, generating sections I–IV. The sections were then stained with hematoxylin and eosin (H&E; Klinipath, Duiven, NL; Merck, Darmstadt, Germany) and Masson's trichrome (Polysciences, Hirschberg an der Bergstrasse, Germany).

The histology staining and assessment of lesions was the main method used for the confirmation of methodological transfer. To determine if the bleomycin instillation reached the trachea optimally, histology slides were initially evaluated qualitatively. Those slides appearing as healthy and normal lung tissue sections, even though they belonged to the bleomycin group (*n* = 2, rats 3 and 8), were considered as non-optimal instilled bleomycin administration and these rats were removed from the study. The remaining histology slides were included in the final evaluation for quantitative assessment, evaluated by two observers for each sample, and scored independently and blindly, using the modified Ashcroft score scale (40). Scoring was performed manually using a microscope and assessed over the entire FOV of the tissue sections. The final mean score from the whole section was presented for each left lung section (I–IV) of each rat, at termination week 1, 2, or 3. Total scores were presented graphically, and representative images were shown from one control and one bleomycin-challenged rat.

Further on, paraffin-embedded and sectioned tissue slices from Group 2 were stained for FAP. Tissue sections were deparaffinated and rehydrated, followed by antigen retrieval (1× TBE + 0.05% Tween-20, 10 min, 96 °C) and peroxidase blocking using a 3% H_2_O_2_. After washing, the sections were blocked with endogenous Biotin/Avidin (Vector, Newark, CA, USA) and 20% Normal Goat serum (Bodinco, Alkmaar, The Netherlands), followed by 60 min of incubation with anti-FAP antibody at RT (1:200 dilution from Abcam, Cambridge, UK; Ab207178). After washing, sections were incubated with a biotinylated secondary anti-rabbit IgG antibody (Vector, Newark, CA, USA) and subsequently Biotin/Streptavidine Complex (Vector, Newark, CA, USA). The staining was visualized with 3,30-diaminobenzidine (DAB) (Immunologic, Arnhem, The Netherlands) and counterstained with H&E (Klinipath, Duiven, The Netherlands). Stained sections were dehydrated and mounted using Permount (Fisher Scientific, Waltham, MA, USA).

### Autoradiography

Formalin-fixed and paraffin-embedded left lung lobe sections (4 μm) from Group 2 were exposed to a Fujifilm BAS cassette 2025 (Fuji Photo Film) for 7 days. Phosphor-luminescent plates were scanned using a phosphor imager (Typhoon FLA 7000; GE, Boston, MA, USA) at a pixel size of 25 μm × 25 μm. Images were analyzed with Aida Image software (version 5.1 SP 6; Elisia-raytest GmbH, Straubenhardt, Germany).

### Statistical analysis

All data were tested for statistically significant differences using statistical tests with the software GraphPad PRISM version 9.03 (GraphPad Software, San Diego, CA, USA, www.graphpad.com). The one-way ANOVA test was applied to identify differences between the groups with *post hoc* testing using Bonferroni's multiple comparisons test. The Student's *t*-test/two-tailed Mann–Whitney test was used to compare the variance between the controls and bleomycin group.

All data are expressed as mean values and SEM unless otherwise specified and *p*-values <0.05 were considered statistically significant. Significance was indicated by * when *p* < 0.05, *p* < 0.01 by **, *p* < 0.001 by ***, and *p* < 0.0001 by **** when comparing the bleomycin-exposed group to the corresponding controls. The comparison between various time points of the bleomycin-exposed group was expressed as # when *p* < 0.05, ## when *p* < 0.01, ### when *p* < 0.001, and #### when *p* < 0.0001.

## Results

### Model characterization and confirmation of model reproducibility

**Figure 2:**
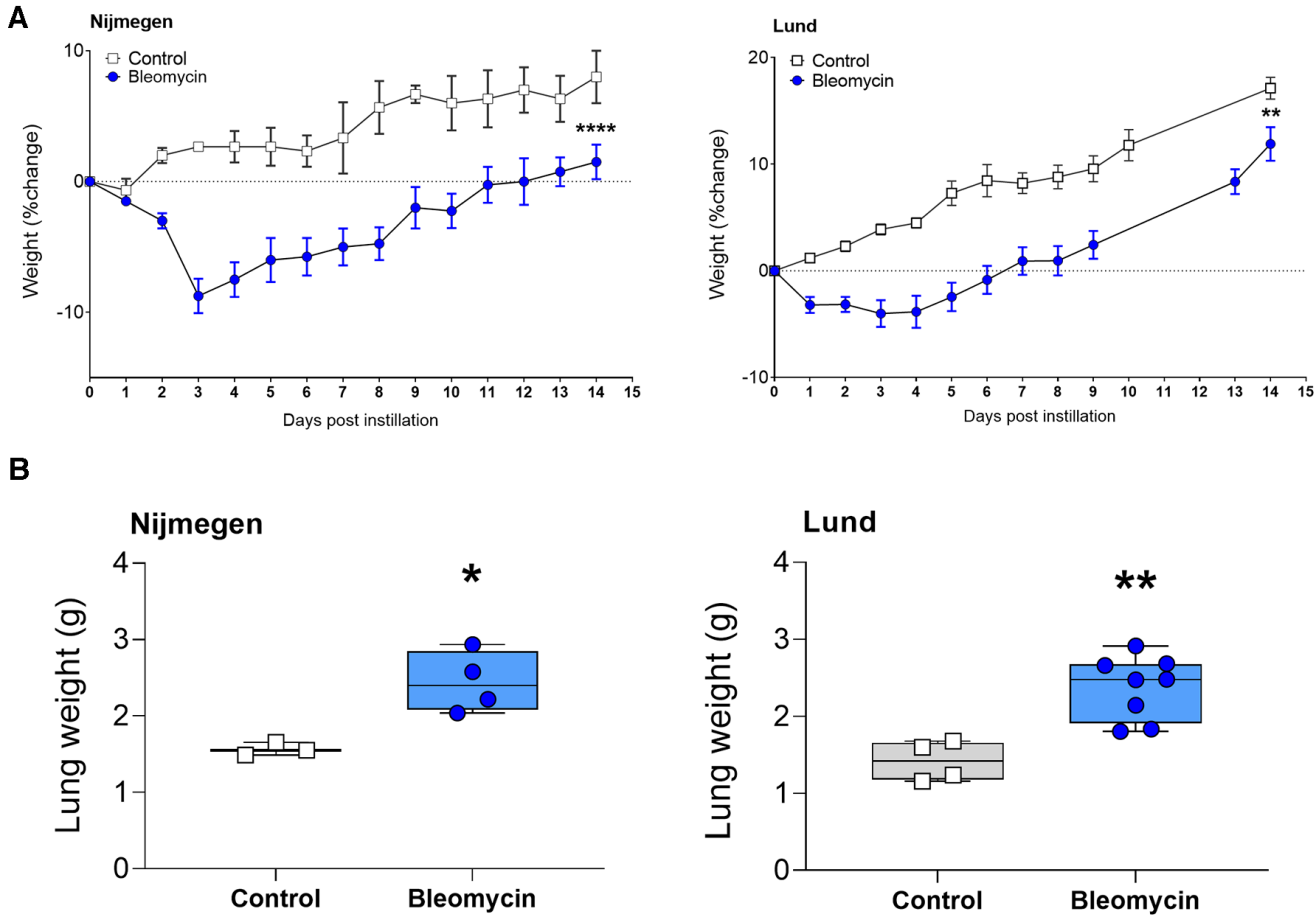
Total body weights and total lung weight at termination, 2 weeks postbleomycin instillation. (**A**) Total body weight of control rats and bleomycin-exposed rats, 2 weeks after i.t. instillation. The percentage of weight change is shown as mean bodyweight per group. (**B**) Lung weight assessed after termination, 2 weeks postinstillation. The weight of each individual rat is shown.

The body weight of the rats dropped during the initial 3–5 days after instillation and thereafter steadily increased again (Figure 2A). The lung weight measured 2 weeks after bleomycin exposure was significantly increased (*p* < 0.05) when compared with that of the controls. Body weight and lung weight followed the same trend at both research sites (Figure 2B) (29).

**Figure 3:**
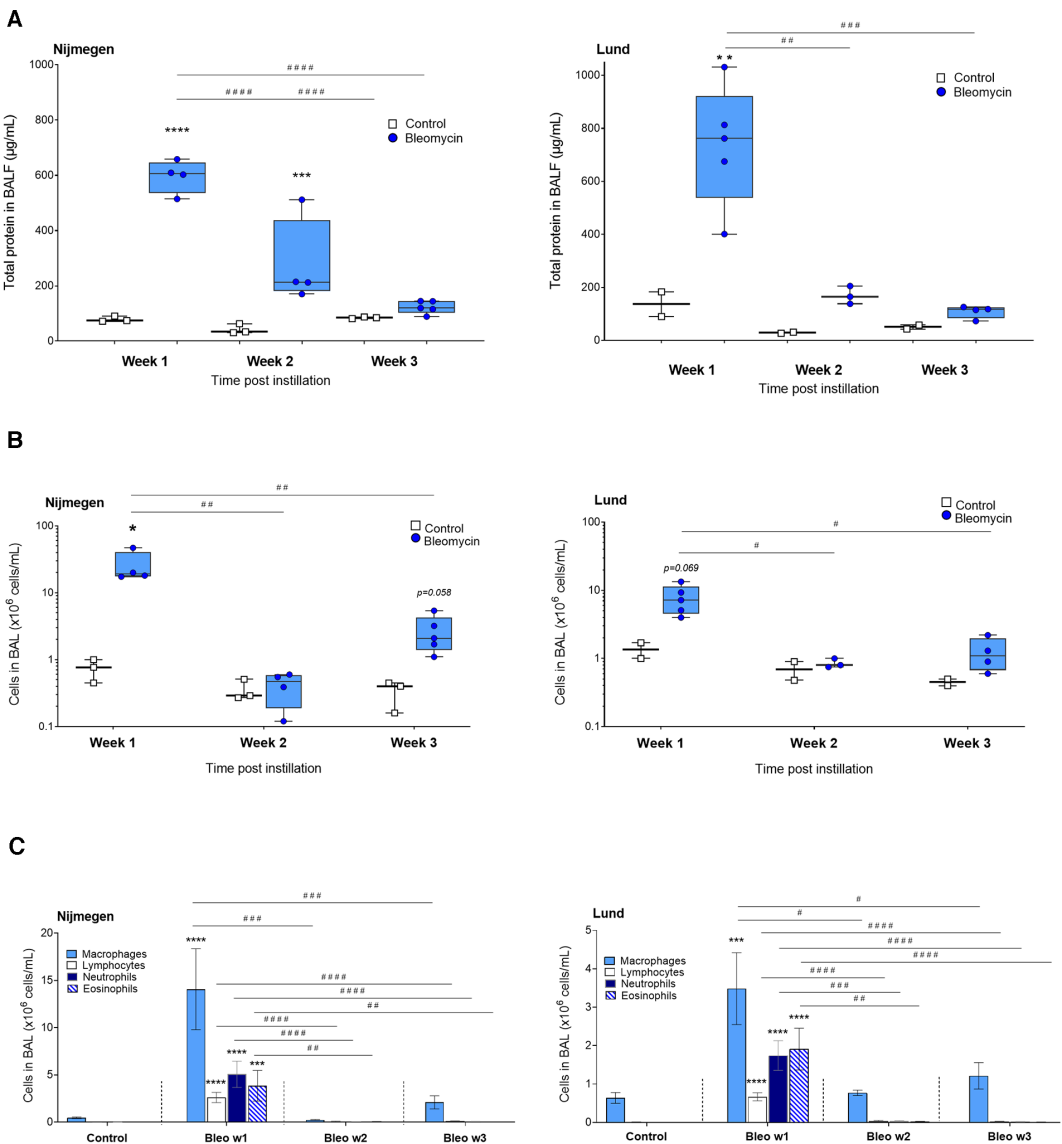
Assessment of total protein concentration and cell counts in BALF at week 1, 2 and 3 postinstillation. (**A**) Total protein concentration determined from BALF supernatants using Bradford protein assay. (**B**) Total cell counts from BAL, presented as million cells per mL. (**C**) The differential cell counts counted from cytospin, as total amounts, are shown from each time point throughout the study.

At 1 week after induction, the total protein concentration in BALF was significantly increased in the bleomycin-exposed rats compared with the control rats (*p* < 0.0001) and subsequently decreased over time (Figure 3A). The total cell counts in BALF were highest in the bleomycin group that was terminated at 1 week after the instillation of bleomycin. The cell counts were decreased by week 2 and started to slightly increase again at week 3 after bleomycin instillation (Figure 3B). The differential cell counts indicated a mixed leucocyte infiltration of the lungs during the first week after bleomycin instillation, with macrophages being the most abundant cell type (Figure 3C). The total distribution ratio of the cell counts from BALF showed a drop of macrophages from 99% (at baseline) down to approximately 50% at week 1, as the other immune cell percentages increased and macrophage counts then returned to similar percentages in week 3, as observed at baseline (Supplementary Figure S2). Overall, the protein content, total cell counts, and differential cell count followed a similar pattern at both research sites (Figures 3A–C) (29).

### Histological analysis of bleomycin-induced lung injury; confirmation of model transfer

**Figure 4:**
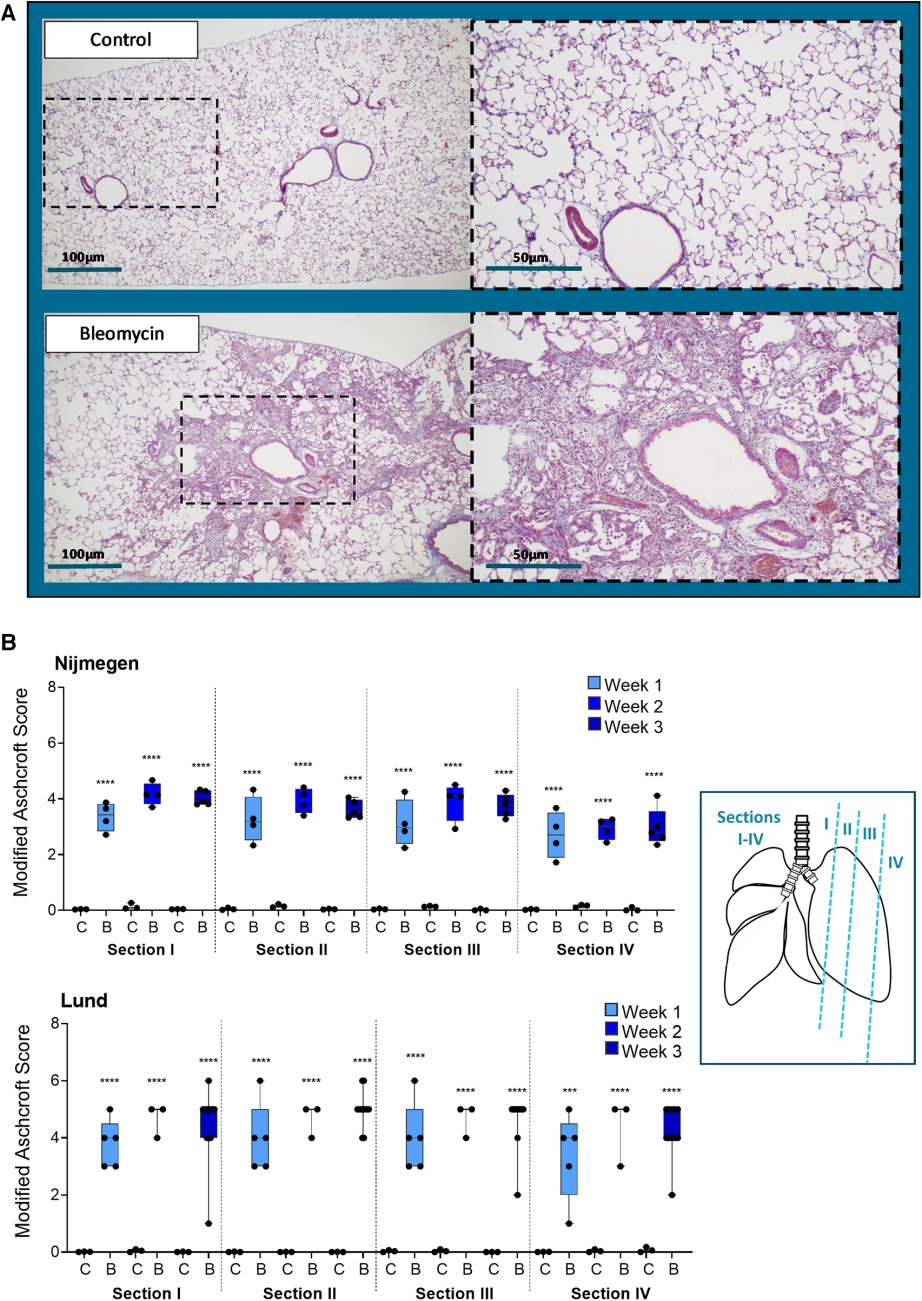
Histology assessment by staining and fibrotic scoring. (**A**) Representative images from Masson's-trichrome stained lung tissue sections, at 2 weeks postchallenge with i.t. administration of saline (control) or bleomycin. (**B**) Histology sections were assessed at four positions (section I–IV), at three different time points in the lungs of control animals (abbreviated as C) or bleomycin-exposed animals (abbreviated as B) by applying the modified Ashcroft score and presented as mean ± SEM.

Representative images of lung tissue sections indicate the presence of lesions induced by i.t. administered bleomycin, compared with controls who received saline i.t. (Figure 4A). The Ashcroft scoring was performed blinded and showed a significantly increased score in the bleomycin group compared with the controls (Figure 4B). In the Nijmegen study, two out of six bleomycin-exposed rats did not show any signs of alterations or development of lung injury upon instillation. Both rats belonged to the longitudinal imaging study (Group 2) and were excluded from all subsequent analysis (Supplementary Figure S3). In the remaining rats, the lesions seemed evenly distributed throughout the lung sections, as the score was in close range between sections I–IV (Figure 4B). The observed pattern of the Ashcroft score over time, upon i.t. instillation of bleomycin, was similar at the different research sites (29).

### Assessment of total lung volume alterations and lesions by MRI

**Figure 5:**
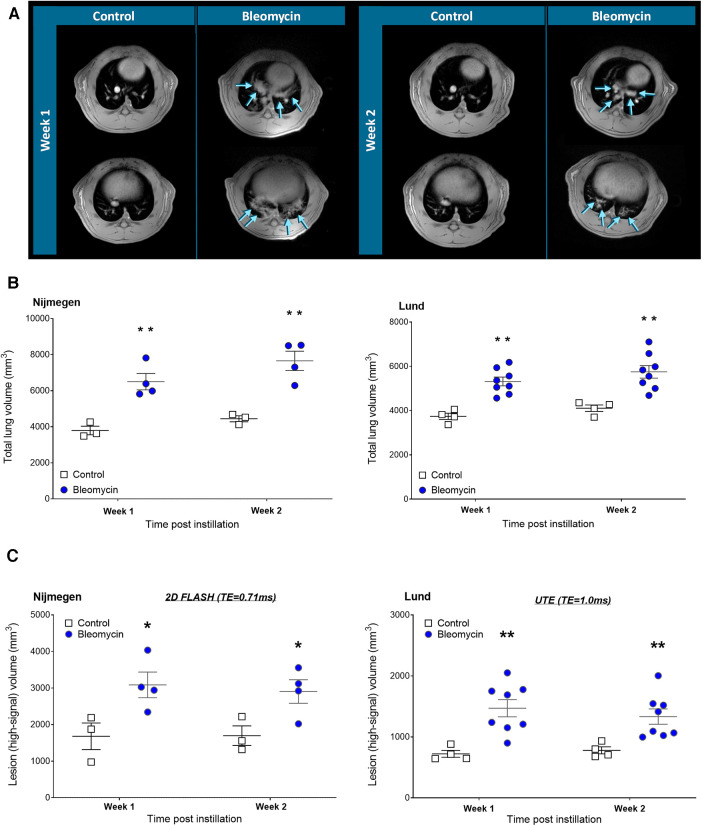
Longitudinal MRI imaging and quantification of total lung volume and lesion volumes. (**A**) Representative 2D transversal slices (showing mid- and lower sections of the lung) after 1 week and 2 weeks postinstillation of saline or bleomycin, at the Nijmegen research site. Arrows point out the location of lesions. (**B**) Total lung volume was assessed by longitudinal MRI scans and (**C**) lesion volume size assessed by extracting the high-signal threshold within the Lung-ROI.

The total lung volume was quantified by assessing the total lung ROI and volume as mean cubic millimeters (mm^3^) for all rats scanned at 1 and 2 weeks after i.t. instillation. Representative transversal 2D MRI slices are presented as examples from one control and one bleomycin-exposed rat at 1 and 2 weeks after the induction of lung injury (Figure 5A). The total lung volume increased significantly (*p* < 0.01) in bleomycin-exposed rats compared with controls (Figure 5B). The assessment of lesions in bleomycin-exposed rats was expressed as the volume of high-signal intensity voxels (Figure 5C). The MRI sequence with a 2D FLASH (TE = 0.71 ms) acquired at the Nijmegen site detected the high-signal voxels, referred to as lesions, in line with the previously used UTE sequence (TE = 1.0 ms) by the Lund site (Figure 5C). The MRI acquisition performed in Nijmegen enabled the detection of a slightly larger volume of high-signal voxels, which could be explained by the shorter TE sequence and lower field strength of the magnet.

### [^18^F]FDG-PET imaging and quantification of lung signal uptake

**Figure 6:**
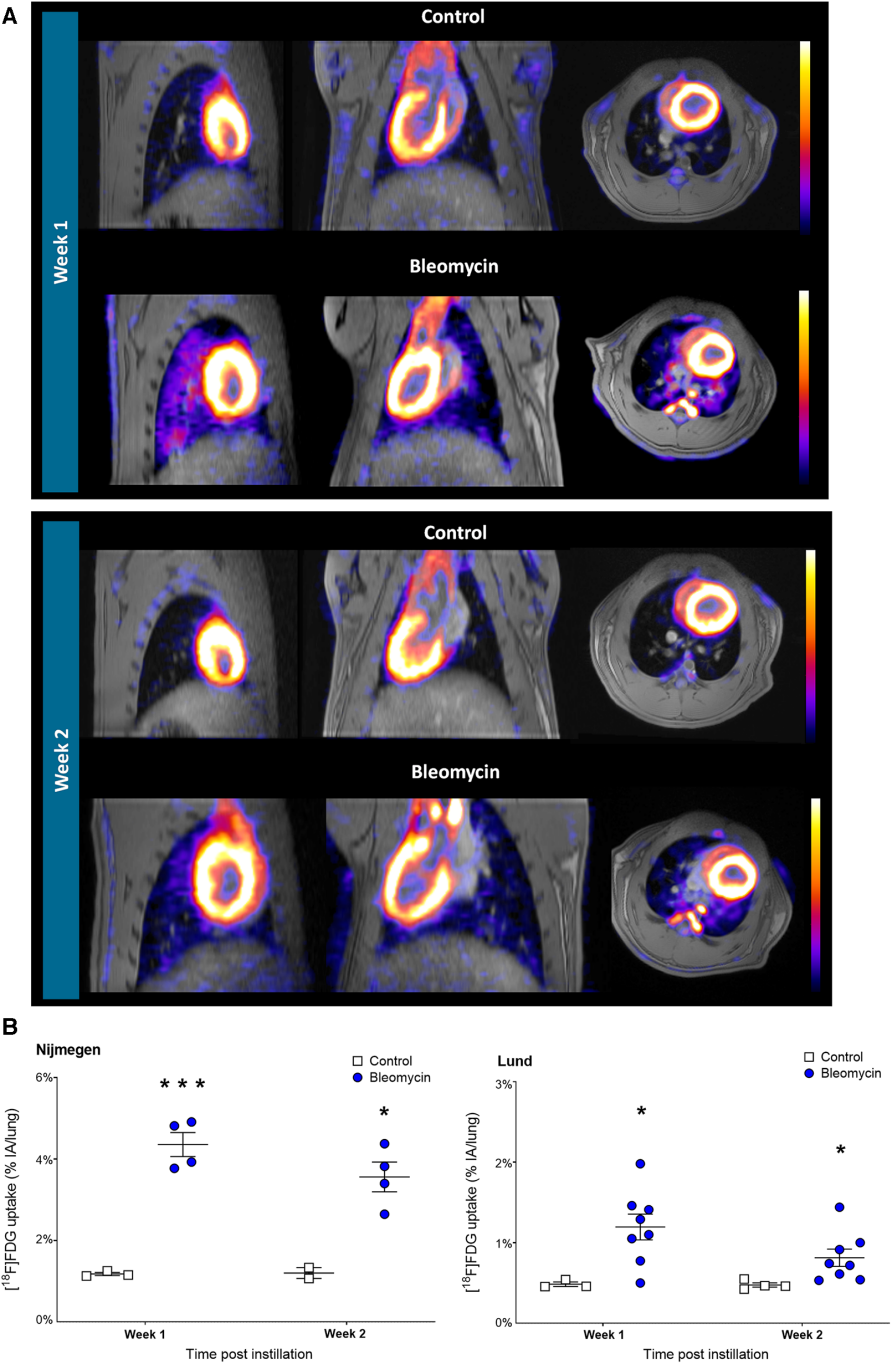
Longitudinal [^18^F]FDG-PET imaging and quantification of lung uptake. (**A**) Representative [^18^F]FDG-PET and MRI images from lungs shown in controls and bleomycin-exposed rats (1 h postinjection of 30 MBq [^18^F]FDG) shown at 1 and 2 weeks postinstillation. (**B**) Comparison of [^18^F]FDG uptake in rat lungs. Data points are expressed for each individual rat.

[^18^F]FDG uptake was assessed at 1 and 2 weeks after i.t. instillation of bleomycin or saline (control). Representative [^18^F]FDG images of bleomycin-exposed rats show uptake in the lungs and heart, while the control animals only show uptake in the heart and minimal uptake in the lungs (Figure 6A). [^18^F]FDG uptake in the lung tissue of bleomycin-exposed rats was significantly higher compared with that in control rats, both at week 1 (4.36 ± 0.59 vs. 1.18 ± 0.07 %IA/lung, *p* = 0.0003) and 2 weeks after induction (3.56 ± 0.73 vs. 1.20 ± 0.19 %IA/lung, *p* = 0.013) (Figure 6B). [^18^F]FDG lung uptake in rats exposed to bleomycin showed a trend toward a reduced uptake after 2 weeks compared with the first week, while lung uptake in the control animals remained constant over time (Figure 6B). This is in line with the Lund study, where significantly increased levels of [^18^F]FDG (*p* = 0.05) were found in the lungs of bleomycin-exposed animals compared with controls (both at 1 and 2 weeks after induction (Figure 6B)), and a trend toward a decreased lung uptake from the first to second week for bleomycin-exposed rats was also evident (Supplementary Figure S4A). Despite these similar trends, in absolute values, the [^18^F]FDG lung uptake was higher at the Nijmegen site compared with the Lund site. Blood glucose measurements did not indicate any variation between time points nor a difference between the groups (Supplementary Figure S4B).

### [^89^Zr]Zr-DFO-28H1 FAP imaging, autoradiography, and immunohistochemistry

**Figure 7:**
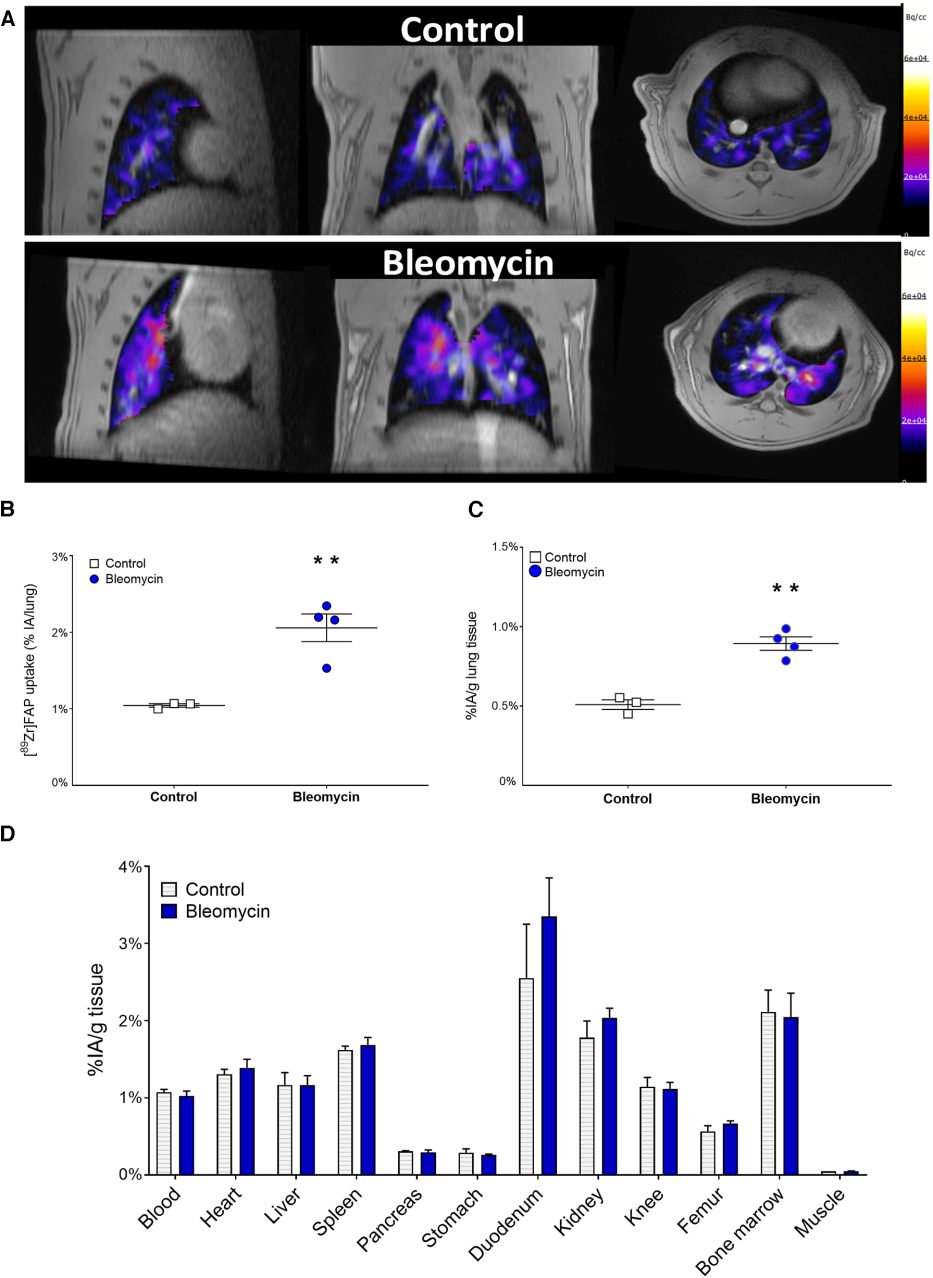
[^89^Zr]Zr-DFO-28H1 FAP imaging, quantification of lung uptake and organ biodistribution. (**A**) Representative [^89^Zr]Zr-DFO-28H1 PET images acquired 24 h postinjection of 5 MBq [^89^Zr]Zr-DFO-28H1. Images from control and bleomycin-exposed rats depict only signal from the Lung ROI for the PET signal. (**B**) [^89^Zr]Zr-DFO-28H1 lung uptake was quantified from the PET imaging and presented as dot-plots where each dot represents one individual rat. (**C**) [^89^Zr]Zr-DFO-28H1 lung uptake quantified from the *ex vivo* biodistribution and (**D**) *Ex vivo* biodistribution data of [^89^Zr]Zr-DFO-28H1 uptake in other relevant organs.

Lung uptake of [^89^Zr]Zr-DFO-28H1 was assessed 15 days after i.t. exposure to bleomycin or saline (control). [^89^Zr]Zr-DFO-28H1 PET showed an increased uptake in the lungs of bleomycin-exposed rats compared with those of control rats (Figure 7A). A quantitative assessment of the [^89^Zr]Zr-DFO-28H1 within the lung showed a significantly higher uptake (*p* = 0.0052) in the bleomycin-exposed rats compared with controls (2.06 ± 0.36 vs. 1.04 ± 0.04 %IA/lung, respectively) (Figure 7B). *Ex vivo* biodistribution confirmed this significant difference in lung uptake (*p* = 0.001) between the bleomycin group and the control group (0.89 ± 0.09 vs. 0.51 ± 0.05 %IA/g tissue, respectively) (Figure 7C), while uptake in other organs showed no differences (Figure 7D).

**Figure 8:**
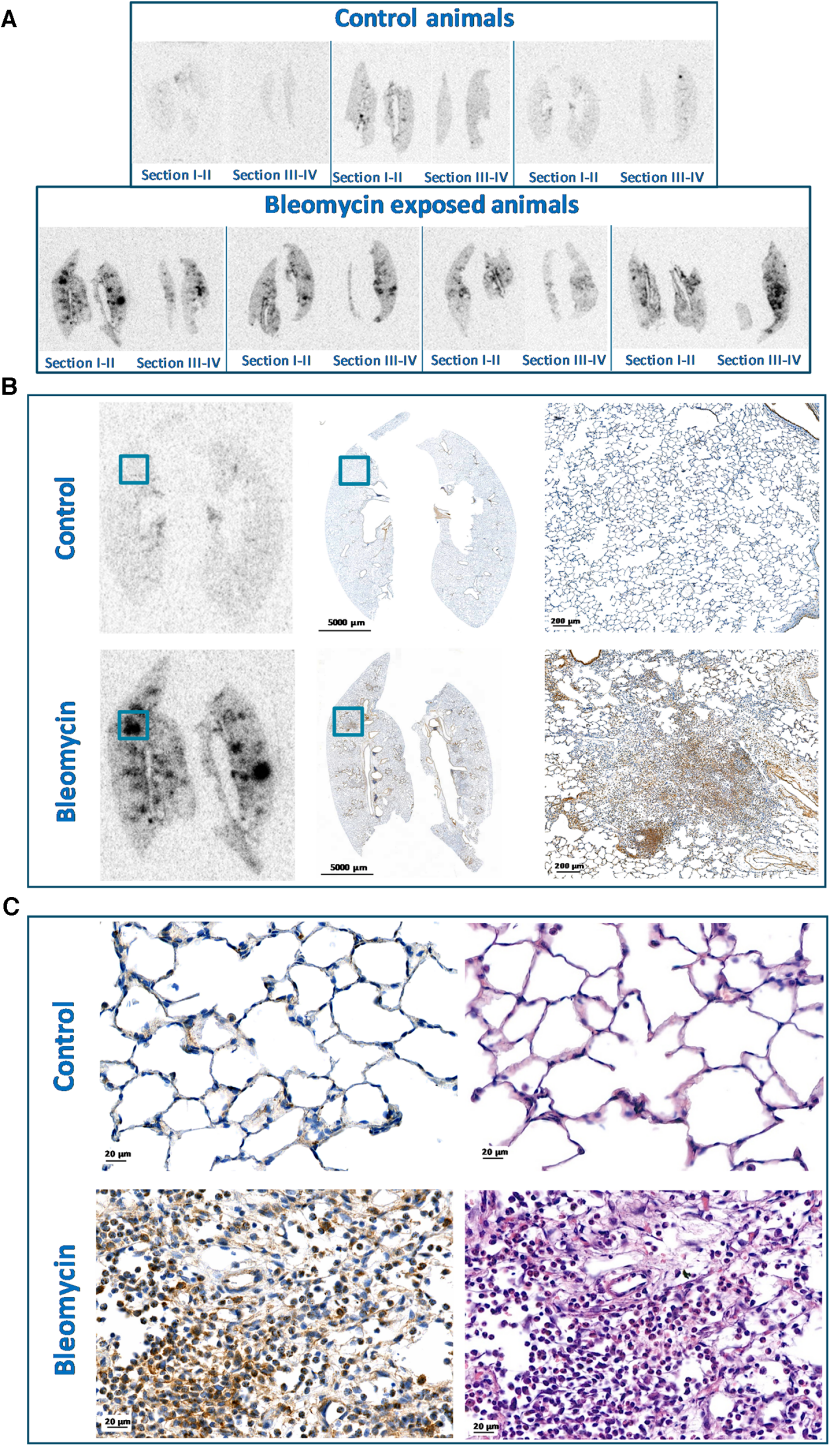
Autoradiography and FAP staining of paraffin embedded lung tissue. (**A**) Autoradiography of [^89^Zr]Zr-DFO-28H1 uptake in paraffin embedded lung tissue section, where all four sections from each rat were included. (**B**) Representative autoradiography images and FAP staining at different magnifications presented from controls and bleomycin-exposed animals. (**C**) H&E and FAP stained lung tissue sections shown from controls and bleomycin-exposed rat lungs. Alteration of the lung tissue is visible in the bleomycin-exposed group with FAP positive cells represented as DAB (brown) stained.

Autoradiographic analyses of lung sections from bleomycin-exposed rats showed high and focal uptake of [^89^Zr]Zr-DFO-28H1, while the uptake in the controls was low (Figure 8A). A comparison of autoradiography images and lung tissue sections showed a high [^89^Zr]Zr-DFO-28H1 signal that colocalized with the presence of FAP-positive cells, as determined by immunohistochemistry, in the bleomycin-exposed lung sections compared to the irregular FAP-positive cells as a background signal from the controls (Figure 8B). Close-up images from lung morphology from bleomycin-exposed rat lungs show areas with high cell densities, containing many FAP-positive cells, indicating the infiltration of immune cells and the presence of activated fibroblasts (recognized by their elongated or spindle like shape) (Figure 8C).

## Discussion

Reproducible lung injury models are key for the successful development of novel imaging biomarkers for early-stage disease detection. Here, we demonstrated that the previously published i.t. bleomycin model (Lund) can be reproduced at a different research site (Nijmegen). Furthermore, monitoring disease progression using imaging biomarkers from MRI and [^18^F]FDG-PET showed similar results at both research sites. Finally, we showed the first proof-of-concept of an FAP-targeting radiotracer to map the early signs of fibrogenesis in this model.

Several methods were used to assess the disease model set-up at both research sites. To identify time-dependent alterations after bleomycin exposure, terminal samples were collected and longitudinal multimodal imaging sessions were performed. Overall, the majority of the data indicated similarities in the disease model at both sites. The various results that matched across both research sites upon bleomycin exposure were loss of body weight, increase in total lung weight, and changes in total protein and cell numbers in BALF. A histological assessment of the lung tissue sections also confirmed that the model was successfully reproduced from one site to another, with clear lesions appearing in the tissues assessed qualitatively and quantitatively using the modified Ashcroft score (40). Even though the histological assessment was done manually, which could introduce room for error and bias, we found that the approach of evaluating each lung at four different positions, including the entire FOV of every lung section (I–IV from each rat), generates the most representative data. For the MRI acquisition, different systems and settings were applied, while the same image analysis method was used (29). The total lung volume increased significantly in the bleomycin groups at both research sites. Lung volume is an important parameter, known to increase after the i.t. instillation of bleomycin, and is a non-invasive imaging biomarker of disease progression in this lung injury model (28, 29, 41, 42).

The two different MRI methods that were used at the research sites were both efficient in terms of detecting lesions within the lung tissue upon bleomycin-induced lung injury. Quantitatively, the lesion volumes were larger in the animals in Nijmegen. These findings are in agreement with the higher [^18^F]FDG-PET uptake also found in these animals. In addition, considering the echo times of the MRI sequences used, in the same order of magnitude, it is expected that the lesions will be somewhat larger when assessed by the 7T MR scanner compared to the 9.4T scanner, due to the different susceptibility effects. Reproducing an MRI protocol on different MR scanners is always challenging, especially when involving both different hardware and software. The aim of this study was to mimic the MRI protocol already in existence in Lund, keeping the difference in resolution between the sites as low as possible. It was not the aim to come up with something completely different (e.g., 3D acquisition), even if that may have resulted in an improvement of the protocol. Even though different hardware and software was used at the two research sites, lesions were still detected and quantified in the bleomycin-exposed lungs, indicating a similar ability for disease assessment.

Tracking disease progression by imaging biomarker [^18^F]FDG-PET showed comparable trends between different research sites, both in terms of the significantly increased [^18^F]FDG uptake in bleomycin-exposed lungs compared with those of the controls, as well as a trend toward lower [^18^F]FDG lung uptake over time, comparing weeks 1 and 2 after the exposure to bleomycin. However, in absolute uptake values, the Nijmegen study showed an almost four times higher [^18^F]FDG uptake. This variability most likely can be explained by the differences in equipment, settings of the detectors, attenuation correction, and postreconstruction methods. This was confirmed by a comparison of the uptake of [^18^F]FDG in the heart of the animals, which also was higher in Nijmegen than Lund (data not shown). However, the differences could also lie in the biological processes, such as higher metabolism in the animals at one of the research sites. Nevertheless, from our studies (e.g., differential cell count and other readouts), we could not identify a single cell type or process responsible for this cause.

The increased uptake of [^18^F]FDG in bleomycin-exposed lungs is a sign of increased metabolism and energy demand in the tissue. However, the exact mechanism and cell types responsible for this increased energy consumption remain to be elucidated (19, 20, 43). During the induction of the lung injury by instillation of bleomycin, the initial inflammation took place and the lung tissue was infiltrated by inflammatory cells. We have shown an increased presence of macrophages, eosinophils, neutrophils, and lymphocytes during the initial 7 days after the bleomycin instillation, which could all contribute to increased energy consumption due to migration and increased cell activity within the tissue. Furthermore, while fibrosis is progressing, activated fibroblasts contribute to the increased metabolism, e.g., by energy-consuming processes, such as the production of ECM components, such as collagen synthesis (19, 27, 44–46). It is most likely a combination of several energy-demanding processes that occurred at the same time, including immune cells present in the initial wound-healing processes as well as active fibroblasts, contributing to the increased metabolism and subsequent [^18^F]FDG uptake (46). Therefore, if we aim to decipher the exact disease mechanisms, more specific radiotracers are needed that visualize a specific target or immune cell population. In the case of lung disease, FAP-targeting tracers, such as [^89^Zr]Zr-DFO-28H1, are of specific interest because these enable the visualization of fibroblasts and, therefore, can be used to track fibrogenesis, one of the key disease processes in lung disease.

Our study shows a twofold increase in lung uptake of the FAP tracer [^89^Zr]Zr-DFO-28H1 in bleomycin-exposed animals compared to controls at 15 days after induction. Autoradiography showed a heterogeneous distribution of the tracer within the lungs. Hotspots of tracer uptake colocalized with the expression of FAP, as assessed by immunohistochemistry. Similar results were found previously using a small molecule FAP inhibitor (FAPI-46) in a bleomycin mouse model (47). A 2.6- and 4.0-fold higher uptake of [^68^Ga]Ga-FAPI-46 was observed at 7 and 15 days after the bleomycin challenge compared with the control animals, respectively. FAP imaging has also been evaluated in human subjects with fibrotic interstitial lung disease with suspected lung cancer (48). It was reported that [^68^Ga]Ga-FAPI-46 uptake showed a positive correlation with the CT-based fibrosis index, suggesting that FAP imaging could be a promising tool to monitor fibrosis. The relevance of FAP imaging has also been shown in patients with inflammatory diseases. Bergman et al. found an increased uptake of [^68^Ga]Ga-FAPI-04 in fibrotic areas of the lungs in patients with systemic sclerosis-associated interstitial lung disease, compared with controls (49). Taken together, both experimental and clinical studies have demonstrated the feasibility of FAP imaging in fibrotic lung disease (50, 51). In the clinical setting, the use of small molecule radiotracers such as FAPI-46 or FAPI-04 is probably preferred over 28H1, because of their faster pharmacokinetics, resulting in lower background radioactivity levels and allowing imaging on the same day as the radiotracer injection. However, future prospective clinical studies still have to prove its clinical value in monitoring lung disease progression and treatment response.

The strength of the bleomycin lung injury model is the robustness and ability to reproduce the model set-up across different laboratories, especially since not only the bleomycin dose and administration route will impact on the final outcome in terms of disease severity. Other factors, such as animal strain, diet, animal holding, and cage mates, might impact how severe the bleomycin-induced disease will be. Nevertheless, any batch difference or variation in administration technique will not be detrimental when inducing disease, since it is fairly easy to assess the terminal samples for model confirmation, in terms of histology and BAL analysis at any time point of this model. The bleomycin instillation induces acute inflammation followed by a drop in body weight during the first few days after the induction of lung injury. Thus, this might hamper long imaging sessions with anesthetized animals. In our experiments, multimodal imaging at time points such as day 7 and onwards could be successfully performed and the animals recovered relative quickly. This is a major strength, as the power of longitudinal measurements is invaluable for disease mapping and follow-up.

The reproducibility of an animal model depends on many factors. A source of uncertainty in our model is the operator-dependent experience of the i.t.-instillations. This is illustrated by the fact that we excluded two rats from Group 2 that did not show any signs of disease upon histopathology and BAL analysis, as well as a lack of lesions on MRI scans. It is most likely that the bleomycin was administered via the esophagus instead of the i.t. route. This highlights the importance of accurate i.t. bleomycin instillation. Another challenge in reproducing animal models between institutes and countries is the difference in local guidelines and legislations, for example in the termination criteria. Finally, one of the potentially largest challenges of reproducing models from one research site to another could be the lack of accurate and detailed descriptions of the experimental procedures. Therefore, more transparent protocols and published details on inclusion or exclusion criteria with animals are warranted.

In summary, the commonly used bleomycin-induced lung injury model and two imaging biomarkers (MRI and [^18^F]FDG-PET) were evaluated at two different research sites. The same administration route and animal strain were employed, while the scanners, settings, and software differed between the research sites. Readouts from the terminal samples showed that the model was successfully reproduced, where samples from BALF, such as cell counts and protein concentration as well as histopathological assessment, indicated comparable trends in disease burden and progression between the two sites. Furthermore, monitoring disease progression with the imaging biomarkers MRI and [^18^F]FDG-PET yielded comparable results. However, it must be noted that in absolute numbers some differences were observed between the two sites, e.g., total lung volume or total uptake of the [^18^F]FDG; however, this was not detrimental for the overall disease readout. Finally, the successful transfer of the lung disease model enabled us to explore the potential of a novel PET imaging biomarker [^89^Zr]Zr-DFO-28H1 to image activated fibroblasts, which are highly relevant in fibrogenesis assessment.

In conclusion, the reproducibility of experimental disease models and imaging biomarkers are significant aspects of translational research. Successful reproducibility enables consistent and reliable results that allow for the interpretation and comparison of data across research sites and studies. Taken together, the i.t. bleomycin rat model is reproducible and robust and allows for future research on new therapeutics or biomarkers in lung disease.

## Data Availability

The original contributions presented in the study are included in the article/**Supplementary Material**, further inquiries can be directed to the corresponding author.

## Supplementary material

Supplementary Figure S1

(**A**) Lung regions of interest (ROI) are presented from all slices in the transverse plane from one rat lung. The ROI area is indicated in turquoise. (**B**) The MRI data analysis that was performed within the entire lung ROI generated initial histograms as shown in the figure. The histogram analysis method was carried out by calculating where the extrapolated line of the slope will intersect on the *x*-axis. The intersection at the *x*-axis thus identifies a threshold value between the normal signal intensity vs. high-signal intensity, within the lung ROI. The appearance of the histogram was notably different in shape between the lung ROIs in the control group and those in the bleomycin-challenged rat group.

Supplementary Figure S2

Differential cell counts were generated from cytospined BAL cells that were stained with May-Grünwald and Giemsa stain and scored by two persons. The counted cells are presented as % ratios, at time points 1, 2, and 3 weeks after bleomycin exposure vs. controls.

Supplementary Figure S3

Histological assessment and presentation of lung tissue sections from controls and bleomycin-exposed rats. In addition, the histology sections from the unaffected lungs of bleomycin-exposed rats (*n* = 2) are presented, which were excluded from the Nijmegen study.

Supplementary Figure S4

Weekly presented (**A**) [^18^F]FDG uptake and (**B**) glucose levels measured before every [18F]FDG-PET scan session, plotting each animal as paired data sets. (**C**) Trends of glucose levels shown between week 1 and week 2 after induction, where each data point represents one animal.
