# Supplementary figures and images for: Utilizing MRI, [18F]FDG-PET and [89Zr]Zr-DFO-28H1 FAP-PET tracer to assess inflammation and fibrogenesis in a reproducible lung injury rat model: a multimodal imaging study
Source: Front Nucl Med. 2023 Dec 12;3:1306251. doi: 10.3389/fnume.2023.1306251 (PMC11440995; doi:10.3389/fnume.2023.1306251)

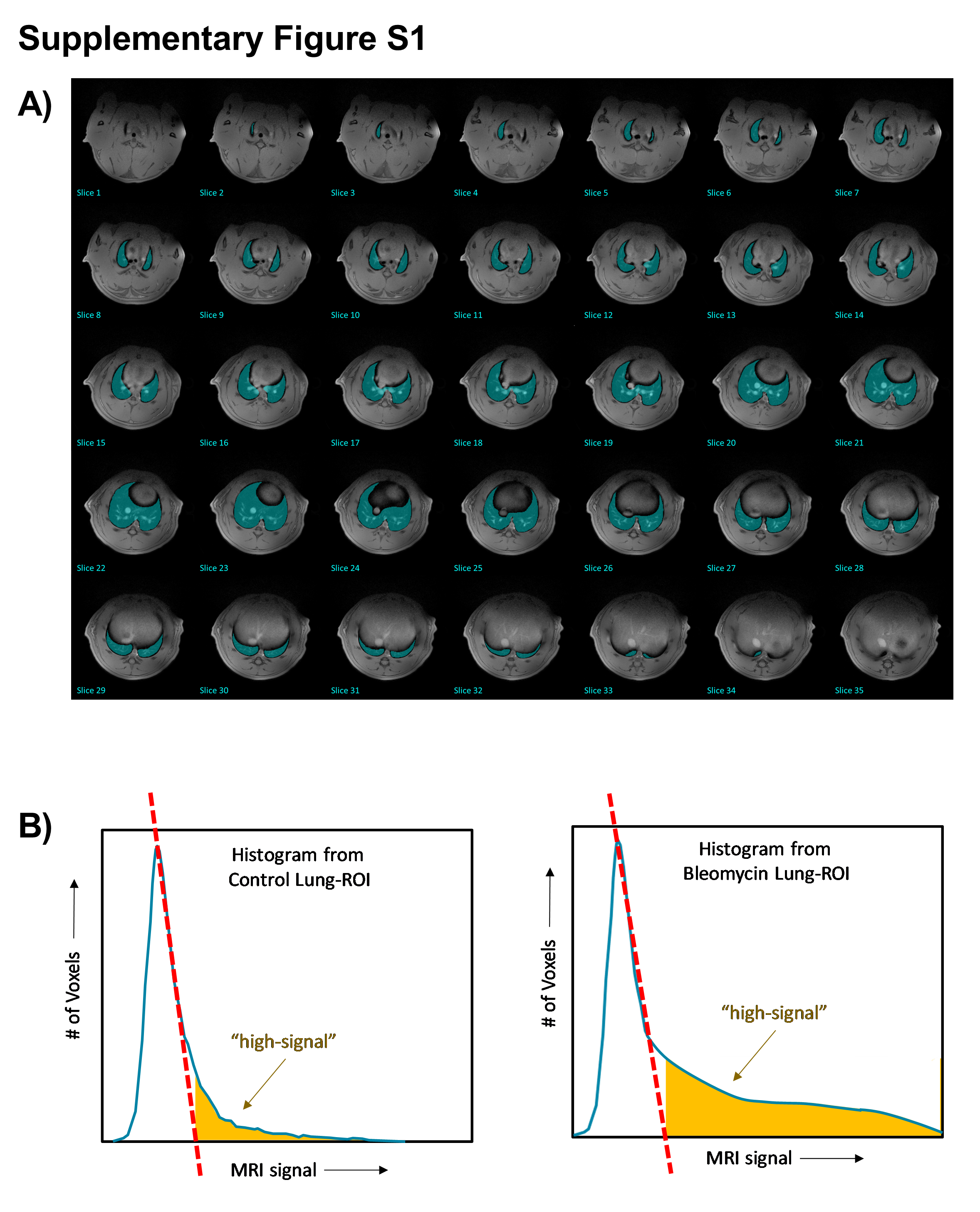

Supplement: Supplementary file 1 [file Image1.tif]

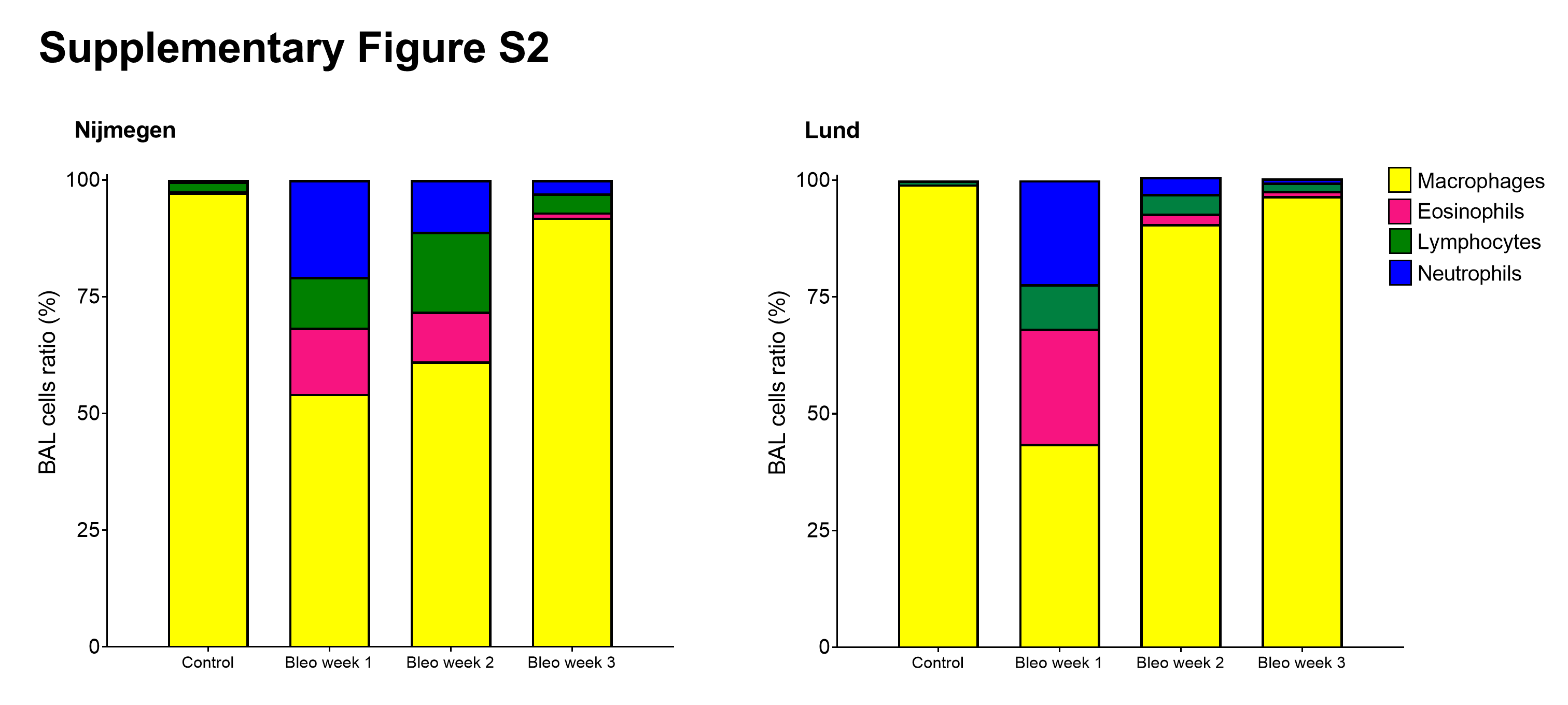

Supplement: Supplementary file 2 [file Image2.tif]

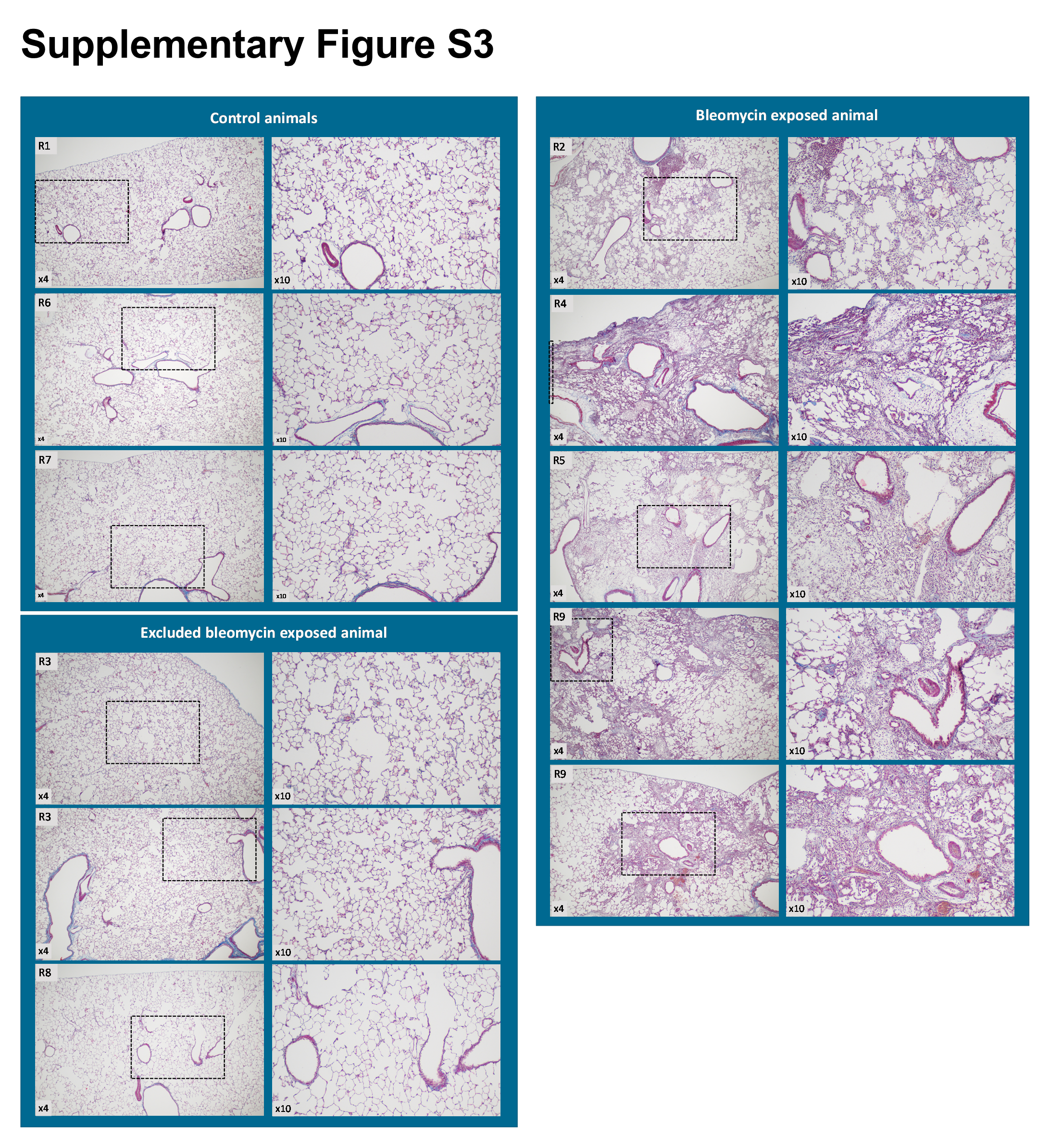

Supplement: Supplementary file 3 [file Image3.tif]

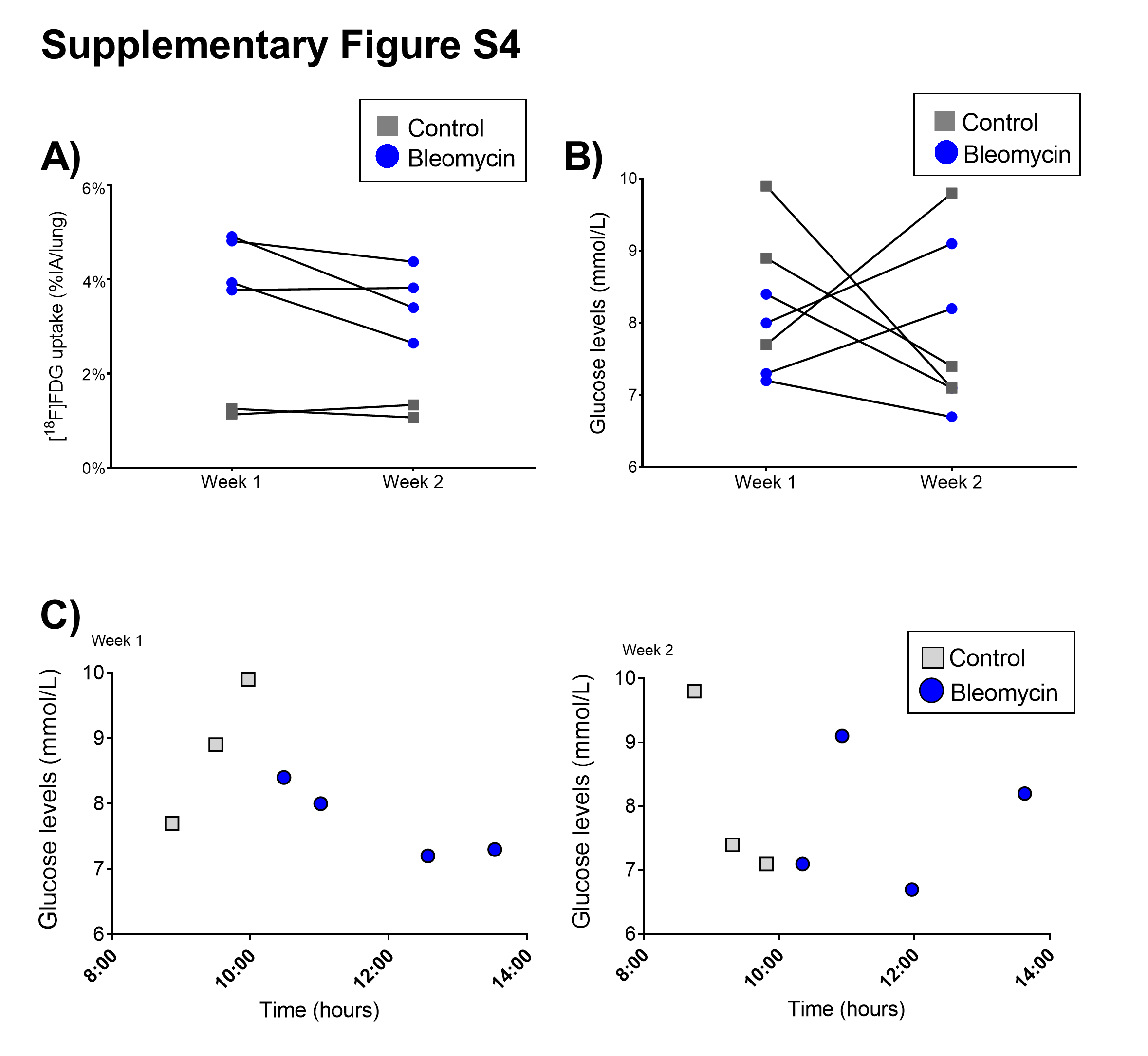

Supplement: Supplementary file 4 [file Image4.tif]
